# The efficacy of continuous-flow cryo and cyclic compression therapy after hip fracture surgery on postoperative pain: design of a prospective, open-label, parallel, multicenter, randomized controlled, clinical trial

**DOI:** 10.1186/s12891-016-1000-4

**Published:** 2016-04-08

**Authors:** Nick C. Leegwater, Peter A. Nolte, Niels de Korte, Martin J. Heetveld, Kees J. Kalisvaart, Casper P. Schönhuth, Bas Pijnenburg, Bart J. Burger, Kees-Jan Ponsen, Frank W. Bloemers, Andrea B. Maier, Barend J. van Royen

**Affiliations:** Department of Orthopedics, Spaarne Gasthuis, Hoofddorp, The Netherlands; Department of Surgery, Spaarne Gasthuis, Hoofddorp, The Netherlands; Department of Surgery, Spaarne Gasthuis, Haarlem, The Netherlands; Department of Geriatrics, Spaarne Gasthuis, Haarlem, The Netherlands; Department of Orthopedics, VU University Medical Center, Amsterdam, The Netherlands; Department of Surgery, Section of Traumasurgery, VU University Medical Center, Amsterdam, The Netherlands; Department of Orthopedics, Amstelland Hospital, Amstelveen, The Netherlands; Department of Orthopedics, Noordwest Ziekenhuisgroep, Alkmaar, The Netherlands; Department of Surgery, Noordwest Ziekenhuisgroep, Alkmaar, The Netherlands; Department of Internal Medicine, Section of Gerontology and Geriatrics, VU University Medical Center, Amsterdam, The Netherlands

**Keywords:** Hip fracture, Cryotherapy, Induced hypothermia, Intermittent pneumatic compression device, Pain, Analgesia, Opioid analgesics, Morphine, Hemoglobin, Delirium, Length of stay, Functional outcome, Patient-reported outcome, Complications

## Abstract

**Background:**

The number of hip fractures and resulting post-surgical outcome are a major public health concern and the incidence is expected to increase significantly. The acute recovery phase after hip fracture surgery in elder patients is often complicated by severe pain, high morphine consumption, perioperative blood loss with subsequent transfusion and delirium. Postoperative continuous-flow cryocompression therapy is suggested to minimize these complications and to attenuate the inflammatory reaction that the traumatic fracture and subsequent surgical trauma encompass. Based on a pilot study in patients undergoing total hip arthroplasty for osteoarthritis, it is anticipated that patients treated with continuous-flow cryocompression therapy will have less pain, less morphine consumption and lower decrease of postoperative hemoglobin levels. These factors are associated with a shorter hospital stay and better long-term (functional) outcome.

**Methods/design:**

One hundred and sixty patients with an intra or extracapsular hip fracture scheduled for internal fixation (intramedullary hip nail, dynamic hip screw or cannulated screws) or prosthesis surgery (total hip or hemiarthroplasty) will be included in this prospective, open-label, parallel, multicenter, randomized controlled, clinical superiority trial. Patients will be allocated to two treatment arms: group ‘A’ will be treated with continuous-flow cryocompression therapy and compared to group ‘B’ that will receive standard care. Routine use of drains and/or compressive bandages is allowed in both groups. The primary objective of this study is to compare acute pain the first 72 h postoperative, measured with numeric rating scale for pain. Secondary objectives are: (non-) morphine analgesic use; adjusted postoperative hemoglobin level; transfusion incidence; incidence, duration and severity of delirium and use of psychotropic medication; length of stay; location and duration of rehabilitation; functional outcome; short-term patient-reported health outcome; general and cryotherapy related complications and feasibility.

**Discussion:**

This is the first randomized controlled trial that will assess the analgesic efficiacy of continuous-flow cryocompression therapy in the acute recovery phase after hip fracture surgery.

**Trial registration:**

www.trialregister.nl, NTR4152 (23^rd^ of August 2013)

## Background

Hip fractures are one of the most important causes of long-term disability and a significant public health issue [1]. Incidence varies on a global scale [2, 3] but incidence is estimated to vary between 414 and 957 per 100,000 inhabitants in the USA [4]. Due to aging of the population in general a significant increase in hip fracture numbers is expected [5].

Hip fractures are a serious condition associated with high morbidity, one-year mortality rates up to 29 % [2, 4], severe pain [6, 7] and significant decline in functional status [8–11]. Less than 50 % of (semi-) independent living elders return to their pre-fracture habitat [11, 12]. Among the most serious of complications is the onset of delirium, which has a particular high incidence of 45 % in hip fracture populations [13–15]. Possibly because this condition is known to be extremely painful [15–18] and patients with pain are nine times more likely to develop a delirium [18–20] and furthermore take longer to ambulate and consequently, have longer length of stay [9].

Upon admission 40 % of hip fracture patients is anemic [21] and observed intraoperative blood loss is systematically underestimated; up to six-fold in excess in extracapsular fractures [22]. The continued postoperative decline in hemoglobin levels results from, among others, surgical site bleeding [22]. This yields a specifically high transfusion rate in this frail elderly patient category where use anticoagulants or thrombocyte aggregation inhibitors is relatively prevalent [23]. Besides (postoperative) surgical site bleeding, the fracture trauma and subsequent surgical stabilization procedure induce a inflammatory response causing leakage of plasma proteins and migration of inflammatory cells that lead to local peripheral vasodilatation and increased capillary permeability, ultimately causing edema [24]. Edema gradually develops during the first week post surgery, is more pronounced in intertrochanteric fractures and correlates well with reduced knee extension strength and functional performance [25, 26]. In an attempt to reduce edema and postoperative hemorrhaging compressive wound dressings are applied [27–29]. Dynamic pneumatic compressive devices not only reduce blood loss, edema and offer a better (hemo) dynamic profile in the deep venous and lymph system, it also exerts analgesic effects and reduces the inflammatory response when combined with a cryotherapy adjunct [30–35].

In a pilot study on 30 patients in a Dutch teaching hospital an apparatus with continuous-flow cryotherapy combined with intermittent compression was used in the postoperative setting of hip arthroplasty for end-stage osteoarthritis [36]. A trend towards lower visual analogue scale (VAS) pain scores and less morphine use was observed, and patients receiving continuous-flow cryocompression (CFC) therapy had statistical significant less decline in postoperative hemoglobin levels. In two randomized controlled trials evaluating continuous-flow cryotherapy (without a compression adjunct) in 45 total hip [37] and 208 total hip and knee arthroplasty [38] patients, lower pain scores were observed and less morphine was used. Furthermore length of stay was 1.4 days shorter when continuous-flow cryotherapy was applied after hip arthroplasty in 74 patients [39].

Currently all published trials studying CFC therapy focus on semi-elective procedures such as anterior cruciate ligament reconstruction and total knee arthroplasties [40]. To our knowledge, no randomized controlled trial exists evaluating the efficacy of CFC therapy in the acute postoperative recovery phase of hip fracture patients [40]. As hip fracture patients have duplicate trauma, severe pain, fracture site bleeding with related inflammation and associated soft tissue damage they are expected to benefit most from CFC therapy.

### Aim

The aim of the current study is to evaluate the efficacy of CFC therapy on pain in the first 72 postoperative hours of hip fracture patients. The secondary aim is to evaluate the effects on (non-) morphine analgesic use; postoperative hemoglobin level; transfusion incidence; delirium incidence and severity; use of psychotropic medication; length of stay; short-term location and duration of rehabilitation; functional outcome; short-term patient-reported health outcome; general and cryotherapy related complications. Furthermore the feasibility of a cryocompression device on orthopedic/surgery wards is assessed.

We hypothesized that: 1) CFC therapy will lower perceived pain levels and morphine consumption; and 2) will reduce postoperative blood loss and transfusion incidence; and 3) reduced pain by CFC therapy will lead to lower delirium incidence and 4) enhance functional recovery, leading to shorter length of stay in postoperative hip facture patients.

## Methods

### Study design

This study is designed as a prospective, open-label, parallel, multicenter, 1:1 randomized controlled, clinical superiority trial in accordance with CONSORT and SPIRIT guidelines [41, 42]. Eight orthopedic, surgery and/or geriatric departments of three middle sized teaching hospitals and one academic hospital in the Netherlands will participate: The Spaarne Gasthuis, Hoofddorp and Haarlem; Noordwest Ziekenhuisgroep, Alkmaar; Amstelland Hospital, Amstelveen; VU University Medical Center, Amsterdam.

### Randomization

**Fig. 1:**
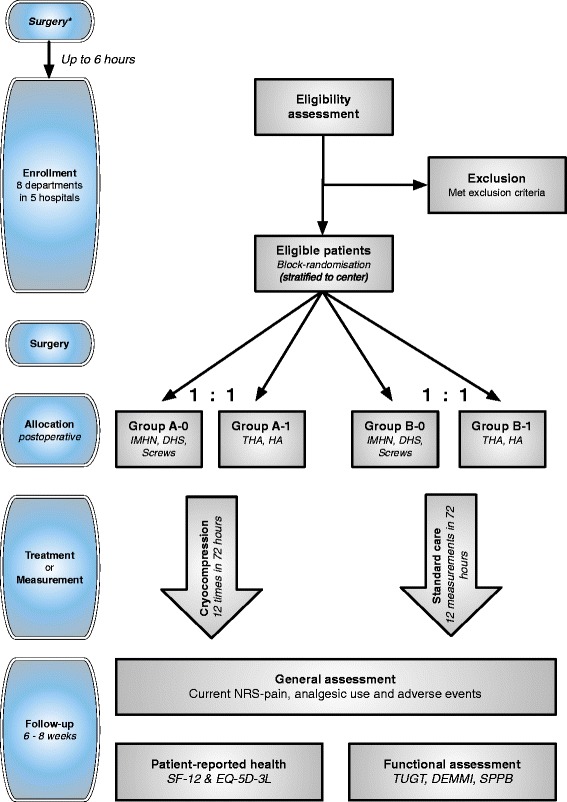
GRAPES-trial flowchart. * Patient enrollment is allowed up to 6 h postoperative. IMHN: intramedullary hip nail; DHS: dynamic hip screw; screws: cannulated screws; THA: total hip arthroplasty; HA: hemiarthroplasty; SF-12: Short Form-12; EQ-5D-3L: EuroQol; TUGT: Timed Up and Go Test; DEMMI: De Morton Mobility Index; SPPB: Short Physical Performance battery

A balanced 1:1 block (size six) randomization stratified by center will be performed directly after surgery, using Research Manager (RM; version 4.5.0.1), a web-based computer program (Fig. 1). A research nurse who is not involved in the study generates the allocation sequence with RM. Intramedullary hip nail (IMHN), dynamic hip screw (DHS) and cannulated screws are grouped for block randomization, as are total hip arthroplasty (THA) and hemiarthroplasty (HA). The intervention group ‘A’ will receive CFC therapy postoperative; the control group ‘B’ will receive standard care. Due to the nature of the study no blinding can exist as patients, family and physicians notice the use and settings of CFC therapy. Physicians randomize participants with a digital logon to the web-based system or the physician can contact the coordinating investigator and he will randomize the included participant (see Table 1 for criteria).The Informant Questionnaire for the Cognitive Decline in the Elderly (IQ-CODE) questionnaire is administered when the clinician has doubts about the cognitive state that may compromise CFC therapy sessions or study measurements (Table 1). Ideally a MMSE would be used for this purpose, however due frequently perceived pain at the accident and emergency A&E department and consequently administered narcotic analgesics, the hetero anamnestic IQ-CODE is used instead. The coordinating investigator contacts the participating hospitals on a daily basis by telephone or physically to ensure adequate and appropriate enrollment of participants and completeness of data. A list of excluded patients will be drafted.

### Standard care

On arrival at the accident and emergency (A&E) department patients receive acetaminophen intravenously, diclofenac (if not contraindicated) and morphine subcutaneously or fentanyl intravenously until numerical rating scale (NRS)-pain scores have dropped below 4. Upon admission the local protocol is started (Table 2). At all centers the 'as needed' medication is administered when the NRS-pain score is 4 or higher and no excessive (opioid-induced) sedation is present. Patients who are deemed able are given intravenous patient controlled analgesia (PCA) pumps with morphine (Table 2). In patients older than 70 years the bolus setting is reduced by 50 %. Preferably patients are operated on using spinal anesthesia. In order to put the patient in an upright position for spinal anesthesia administration femoral blocking with short-acting analgesics can be used. All centers use bupivacaine 0.5 % between 2.0 and 3.0 ml administered at the lumbar level. Additional (long acting) analgesics administered during surgery are noted. The participating centers adhere to the Dutch national guidelines for surgical technique for the various fracture types [43]. Postoperatively, on the day of surgery or the first postoperative day physical therapy is commenced once or twice daily. Physical therapy sessions are usually 30 min long. In the period in which the focus of this study lies physical therapy focuses on strengthening of quadriceps and gluteal musculature, walking and making transfers.

**Table 1:** Criteria for participants in the trial

Inclusion criteria	Exclusion criteria
* Intra or extracapsular proximal femur fracture	* Fractures at multiple foci
	* Open fracture/wounds^a^
* Aged 18 years and over	* Acetabular fracture
* Informed consent or proxy consent	* (Suspicion of) concomitant malignancy
	* BMI > 40
	* Unwilling to give proxy consent
	* Preoperative osteosynthesis materials in situ in the ipsilateral leg above knee level
	* Morphine allergy or dependence
	* ASA ≥ 4
	* Cryoglobulinemia
	* M. Raynaud
	* Central neuromuscular disorder
	* Absent ADP/ATP pulsations in the injured extremity
	* Active deep vein thrombosis
	* Suspected pulmonary embolism
	* Patient delay > 24 h
	* NYHA-class ≥ 3
	* IQ-CODE score ≥ 4.6*
	* Long-acting femoral blocks
	* Use of LIA
	* Postoperative HD-instability

^a^Open wounds unable to close per primam, * The IQ-CODE is only administered if the clinician has doubts about the cognitive status of the intented participant

*BMI* body mass index, *ASA* American Society of Anesthesiologists, *ADP* dorsal pedal artery, *ATP* posterior tibial artery, *NYHA* New York Heart Association, *IQ-CODE* Informant Questionnaire for the Cognitive Decline in the Elderly, *LIA* local infiltration anesthesia, *HD* hemodynamic

**Table 2:** Hospital protocols

		Analgesic protocol				
Hospital	Thrombosis prophylaxis	Standard		As needed		
Spaarne Gasthuis, Hoofddorp	Fraxiparine 2850 IU 1x^a^	Acetaminophen 1000 mg 4x	Diclofenac 50 mg 3x^b^	PCA-iv morphine	*or*	Morphine 10 mg 6x sc^c^ *or* Oramorph 10 mg 6x^c^
				*If considered able*		
Noordwest Ziekenhuisgroep	Fraxiparine 2850 IU 1x^a^	Acetaminophen 1000 mg 4x	Diclofenac 50 mg 3x^b^	PCA-iv morphine	*or*	Morphine 10 mg 6x sc^c^ *or* Oxycodone 10 mg 6x^c^
				*If considered able*		
VU University medical center	Fraxiparine 2850 IU 1x^a^	Acetaminophen 1000 mg 4x	Diclofenac 50 mg 3x^b^	Morphine 10 mg 6x sc^c^	*or*	Oxycodone 10 mg 6x^c^
Spaarne Gasthuis, Haarlem	Fraxiparine 2850 IU 1x^a^	Acetaminophen 1000 mg 4x	Diclofenac 50 mg 3x *or* 75 mg iv 2x^b^	Morphine 10 mg 6x sc^c^	*or*	Oxycodone 10 mg 6x^c^
Amstelland Hospital	Enoxaparine 40 mg 1x	Acetaminophen 1000 mg 3x	Diclofenac 75 mg im/iv 2x^b^	PCA-iv morphine	*or*	Piritramide 10 mg 6x sc^c^ *or* Tramadol 50 mg 3x
				*If considered able*		

Reported dosages are dosages given in 24 h

*im* intramuscular, *iv* intravenous, *sc* subcutaneous, *PCA* patient controlled analgesia

^a^Dosage is doubled in patients weighing > 80 kg

^b^If no contraindications exist. ^c^: Dosage is reduced by 50 % in patients aged ≥ 70 years

### Study apparatus and treatment schedule

**Fig. 2:**
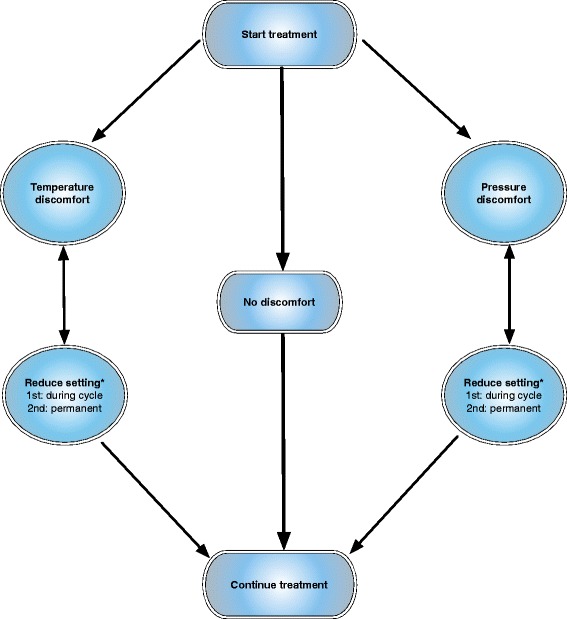
Discomfort flowchart. * If a patient reports discomfort for the second time at the same setting this setting is permanently reduced to the setting deemed comfortable

Continuous-flow cryocompression therapy is applied by using the ‘Game Ready system’ (GRS; CoolSystems Alameda, California). Through an anatomically designed hip/groin wrap covering most of the thigh and pelvis up to the iliac crest, the GRS simultaneously delivers both adjustable continuous-flow cryotherapy and intermittent compression through a portable control unit filled with ice and water. The machine has four pressure settings: no pressure, low pressure (5–15 mm Hg), medium pressure (5–50 mm Hg) and high pressure (5–75 mm Hg). Temperature can be adjusted between 4 and 13 °C and is indicated by one, two or three snowflakes. The lowest temperature is started and maintained throughout the study if feasible. Pressure is started at ‘low’ and is increased one step per 4 treatments (Table 3). Depending on the end of surgery patients will be categorized in three treatment schedules with respective start and end-times (Table 3). If patients are uncomfortable the appropriate adjunct will be adjusted stepwise until a comfortable setting is reached, deviations are noted. Adjustments are recorded and comfortable settings are maintained according to the discomfort flowchart (Fig. 2). Patients will be treated between 10 and 12 times during the first 72 postoperative hours, each cycle lasting 30 min. Preferably, treatment cycles and control measurements during the first 72 postoperative hours are performed at fixed moments: 8:00 h, 12:00 h, 16:30 h, and 21:30 h. The GRS-wrap is only in place when CFC therapy is administered and applied/removed by the nurse.

**Table 3:** Treatment schedule and pressure settings

End time surgery	Schedule	Start time										
10:00 – 16:00 h	*A*	16:30 h										
16:00 – 21:00 h	*B*	21:30 h										
>21:00 h	*C*	8:00 h										
Cycle no. →	1	2	3	4	5	6	7	8	9	10	11	12
OK + 0	*A:* – Low	*A* – Low										
	*B: –* Low	-										
	-	-										
OK + 1	-	*-*	*A* – Low	*A* – Low	*A* – Med	*A* – Med						
	-	*B –* Low	*B –* Low	*B –* Low	*B –* Med	-						
	*C* – Low	*C* – Low	C – Low	*C* – Low	-	-						
OK + 2					*-*	*-*	*A* – Med	*A* – Med	*A –* High	*A –* High		
					*-*	*B –* Med	*B –* Med	*B –* Med	*B* – High	-		
					*C –* Med	*C –* Med	*C –* Med	*C –* Med	-	-		
OK + 3									-	*-*	*A –* High	*A –* High
									-	*B* – High	*B* – High	*B* – High
									*C –* High	*C –* High	*C –* High	*C –* High

Regular treatment times: 8:00 h; 12:00 h; 16:30 h; 21:30 h

**Table 4:** Outcome assessment points

		Assessment point						
Item	Data collection instrument (unit)	Baseline (A&E department)	Day of surgery	24 h	48 h	72 h	Discharge	6 - 8 weeks (outpatient clinic visit)
Demographics	-	X						
Pre-fracture functional status	NMS, KATZ-ADL	X						X
Delirium risk assessment	DRAS	X						
Cognitive function	IQ-CODE	X						
	MMSE						X	X
Blood loss	Intraoperative loss *(cc’s)*		X					
	Drain output^a^ *(cc’s)*			X	X	X	X	
Outcome								
Pain	NRS	X	X	X	X	X	X	X
Analgesics	Acetaminophen	X				X	X	
	NSAID’s	X				X	X	
	Morphine	X				X	X	
Blood loss	Hemoglobin (mmol/l)	X		X		X		
Transfusion incidence	Number of units						X	X
Delirium	DOS-score	X	X	X	X	X	X	
	DRS-R-98^c^		X	X	X	X	X	
	Pyschotropic medication	X				X	X	
Length of stay^b^	*(Hours)*						X	
Functional outcome	TUGT						X	X
	DEMMI							X
	SPPB							X
	Rehabilitation location						X	X
	Rehabilitation duration						X	X
PROM	EQ-5D-3L							X
	SF-12							X
	Satisfaction questionnaire						X	
Complications	General						X	X
	Therapy related						X	X
Feasibility	Nurse staff questionnaire						At discharge of last patient	

*A&E* accident and emergency, *NMS* New Mobility Score, *IQCODE-N* Informant Questionnaire on the Cognitive Decline in Elderly patients, *MMSE* Mini Mental State Exam, *NRS* numeric rating scale, *PC* packed cells, *DOS* Delirium Observation Screening, *DRS-R-98* Delirium Rating Scale Revised 98, *TUGT* Timed Up and Go Test, *DEMMI* De Morton Mobility Index, *EQ-5D-3* *L* EuroQol questionnaire, *SF-12* Short Form-12 questionnaire

^a^Including autologous reinfusion. ^b^Start count at admission and at the end of surgery. ^c^: administered when DOS ≥ 3

### Co-interventions

In case of discomfort the appropriate adjunct is adjusted accordingly (Fig. 2) and supplemental analgesics are given as needed (Table 2). In both the intervention group receiving CFC therapy and in the control group no restrictions are made towards regularly used static compressive bandages and/or wound drains (with or without autologous re-infusion). Geriatricians are consulted in a standard fashion in patients aged 70 years and over.

### Admission

Patient demographic data are noted on admission as well as American Society of Anesthesiologists (ASA) class, current NRS-pain, delirium observation screening (DOS)-score, activities of daily living (ADL)-Katz score and the New Mobility Score (NMS; Table 4) [44–46]. Time of injury and arrival at the A&E department is noted. Patient delay intervals longer than 6 h are actively explored and reasons for delay are clarified e.g. if a patient was prone and unable to call emergency services.

A standard preoperative venous blood sample is taken in which hemoglobin, hematocrit and mean corpuscular volumes values are measured (Table 4). Upon admission the local hospital analgesic and antithrombotic protocol is started (Table 2). A geriatrician is consulted preoperatively in patients who are older than 70 years and will focus on: polypharmacy, risk reduction of falls, prevention and/or treatment of delirium, optimization of nutritional status. The ADL-Katz score and delirium risk assessment score (DRAS) [47] are completed upon admission; furthermore precipitating factors for delirium (bladder infection and/or retention, fixation, sleep deprivation, electrolyte abnormalities including renal function impairment) are noted throughout the hospital stay. After the operation the surgeon notes the intraoperative blood loss, the type of implant, if applicable intraoperative complications, randomizes the patient and determines the patient’s treatment schedule.

### Cryocompression therapy and clinical measurements

Intervention patients start with CFC therapy 6 h postoperative, until 72 h postoperatively. A trained ward nurse will conduct the pain assessments when the patient is in bed or stationary in a chair for at least five minutes. Directly before and directly after CFC therapy vital signs including NRS-pain are measured by the ward nurse, when the patient is prone in bed for at least five minutes before commencing and five minutes after cessation of CFC therapy. In control patients only NRS-pain is measured at the same time as intervention patients would normally be assessed. All pain measurements are continued throughout the first postoperative 72 h for control and intervention patients alike (Table 4). Three times daily DOS-scores [48] will be obtained; in case of a DOS-score higher or equal to three, geriatricians diagnose and subsequently monitor delirium severity with the delirium rating scale revised 1998 (DRS-R-98) [49] on a daily basis or until the score drops below 12.25 out of 39 (Table 4). Time and amount of administered (psychotropic) medication will be documented. The nurse and ward doctor inspect the wound and dressing in accordance with routine care. Blood samples are taken at postoperative day one and three, and post-transfusion if applicable (Table 4). The timed up and go test (TUGT) is performed before discharge (Table 4). The TUGT will be administered in all patients who are able. If no weight bearing is allowed or patients are physically unable, than the test will be postponed to the outpatient visit. The mini mental state examination (MMSE) is administered for data stratification purposes [50–52], if patients are delirious during admission the MMSE is postponed to the outpatient visit.

In order to document experiences with CFC therapy all treatment patients will fill in a questionnaire at discharge, which is specifically drafted for this study (Table 5). During the study a booklet will be made available into which staff can write down prevalent occurring technical GRS-related problems.

**Table 5:** Patient questionnaire

1. Did you feel that the pain reduced when treated with cryocompression therapy?	A	B	C	D	E
2. Would you rather have cryocompression treatment than analgesic pain treatment?	A	B	C	D	E
3. The standard setting used was the coldest; did you like this temperature setting?	A	B	C	D	E
4. Did you request the temperature setting to be upped?	Y				N
5. Did you like the dynamic pressure adjunct?	A	B	C	D	E
6. The pressure adjunct was elevated every 4 treatments, was the pace to fast?	A	B	C	D	E
7. After treatment the muscles are cooled, did this hinder you in moving around outside of bed?	A	B	C	D	E
8. Would you have liked to be treated more often per day than 4 times?	A	B	C	D	E
9. Would you have liked to be treated longer than 30 min per cycle?	A	B	C	D	E
10. Would you have liked to be treated longer than the first 72 h postoperative?	A	B	C	D	E
11. Did you feel like you recovered faster with cryocompression therapy?	A	B	C	D	E
12. Would you recommend the use of cryocompression therapy to other patients?	A	B	C	D	E
13. Can you briefly describe what you think are advantages of cryocompression therapy?	Open text				
14. Can you briefly describe what you think are disadvantages of cryocompression therapy?	Open text				
15. From 0 – 10 how would you rate the cryocompression treatments in general?	(0-10)				

A: agree completely; B: agree; C: neutral; D: disagree; E: disagree completely

Y: yes; N: no

### Outpatient visit

At the single outpatient study visit between six to eight weeks, the following parameters are assessed; current NRS-pain; analgesic use; mobility status by the TUGT, de Morton Mobility Index (DEMMI) and the Short Physical Performance Battery (SPPB); current living situation (rehabilitation or at home); wound status and complications (thrombosis, consolidation, infection, readmission). Furthermore the self-assessment health questionnaires EuroQol(EQ)-5D-3 L and Short Form (SF)-12 are completed. If not administered during admission the MMSE is completed (Table 4).

### Data collection and handling

Upon inclusion patient characteristics are entered in RM and postoperative randomization takes place. A unique patient number is generated by RM, which will be used for further data collection on preformatted case report forms (CRF’s). Only the coordinating investigator has access to the key-file that can identify patients from RM-generated patient numbers, it will be stored at the coordinating center throughout and after completion of the trial and will not be shared. The Spaarne Gasthuis is the unrestricted owner of the final dataset; no contractual agreements exist in regard to publication policies. No interim analysis will be performed.

### Data management team

The coordinating center comprises of: the principal investigator, the coordinating investigator, a research nurse, a research assistant, a clinical epidemiologist and statistician. The research assistant enters data in RM. The research nurse can be contacted via telephone to solve any (acute) problems that might occur and enters data in RM. The epidemiologist and statistician assist with data analyses and drafting of the manuscript. During the enrollment phase of the trial the coordinating investigator meets bimonthly with the individual principal investigators of the respective centers to assess the current inclusion rate, data quality, protocol adherence and prevalent occurring problems. During enrollment two meetings are planned to collectively assess problems and deliberate on how to instigate solutions to these problems. All participating personnel are updated on a monthly basis via a newsletter in which current inclusion rates are presented, and to point out points of attention and solutions to frequently observed problems. During physical visits CRF’s are collected, assessed for completeness and missing data is retrieved from patient medical records where possible; secondly the appropriateness of administered treatments and (functional) tests is assessed.

### Data monitoring committee

Two independent data monitors are instigated and will monitor the trial at two of the five sites where highest inclusion rates are expected. Results of the monitor will be discussed in a committee independently of the investigators and the investigators will be informed both orally and in writing. Since the overall risk of participation in the trial is considered low by the REC/IRB no external safety overseers are required by national guidelines. If selected, external auditing is possible.

## Outcome parameters

### NRS-pain (primary)

The verbally administered 11-point (range 0–10) NRS-pain is a widely accepted assessment tool and has been validated in many different conditions, among is acute pain in the emergency department [53–55]. Reliability and validity has been thoroughly studied in (cognitively impaired) elderly and has shown to maintain acceptable clinometric characteristics [56–58]. NRS-pain scores are compared within the intervention group i.e. pre and post-treatment and compared between the intervention and control groups. Cumulated pain scores will be calculated for both groups by adding the (post-treatment) pain scores of the first 72 postoperative hours.

### Analgesic use

All postoperative oral and parenteral administered narcotic analgesics will be converted and added up using accepted algorithms [59, 60] and compared after the last treatment day, at discharge and at the outpatient clinic. Total milligrams of acetaminophen and non-steroidal anti-inflammatory drugs (NSAID’s) are reported in the same fashion. At the outpatient visit patients will be asked about their current pain medication use.

### Postoperative blood loss and transfusion incidence

The hemoglobin values from the obtained blood samples at postoperative day one and three will separately be detracted from the preoperative concentration and postoperative day three will be detracted from postoperative day one. An adjustment will be made for the intraoperative blood loss. Indications for transfusion are managed according to the Dutch national blood transfusion guideline [61]. Normovolemic ASA-one patients older than 60 years who lose blood at a single locus will be transfused at 5 mmol/l (8 g/dl). Patients unable to elevate cardiac output for hemodilution are transfused at 6 mmol/l (9.6 g/dl). Incidence of erythrocyte transfusion is noted and compared between groups.

### Postoperative delirium

The DRS-R-98 is considered to be a valid and reliable instrument for delirium diagnosis and documenting delirium severity [49, 62]. It takes a trained nurse or physician five to ten minutes to assess the patient and comprise the DRS-R-98 score, and is ideal for longitudinal delirium follow-up. The sensitivity and specificity of the scale are reported to be 92 % and 86 % respectively. DRS-R-98 scores are compared within and between groups, and newly started psychotropic medication is compared.

### Length of stay

Admission time is measured and expressed in two ways: from arrival time at the A&E department until discharge and from end of surgery to until discharge. The discharge criteria in all participating centers are: NRS-pain score below 5 without need for parenteral analgesics and an Elderly Mobility Scale (EMS) score 14 out of 20 or higher [63, 64]. If this some items were deemed unobtainable EMS scores are completed until transfer to a nursing home is arranged. If family members are able to foresee in certain home care aspects the patient can be discharged to his/her home. The fulfillment of this care by family is noted and corrected for afterwards.

### Functional outcome

#### Timed Up and Go Test

The TUGT is a functional mobility test that measures the time it takes a patient to get up out of a chair, walk 3 m and return in a sitting position. The test is easy to use and has sufficient clinometric characteristics [65], can predict short-term risk of new falls [66] and functional outcome over time [67]. Detailed instructions are provided to the patient: rise from the chair (knees at 90° flexion) when you hear “go”, walk at safe speed to the mark on the floor and back, the time stops when you hit the chair with your buttocks. The physical therapist will demonstrate how to perform the TUGT once. The average of three tests will be the final TUGT outcome and no trial run is performed. Postoperative mobilization policy is noted and patients are stratified in three groups: no weight bearing, partial weight bearing or full weight bearing. Preferably no walking aid will be used during the test but if needed, the patient can use any walking aid available, the type of aid used will be recorded. The updated NMS is administered at inclusion for data stratification purposes [44, 45, 68]. NMS will be classified as low when NMS is 2-6 and scored high when NMS is 7–9 [46].

#### De Morton Mobility Index

The DEMMI is a measure of mobility that has been validated in hip fracture patients [69-71]. The DEMMI is administered by physician or physical therapist observation of the test subject’ physical performance, measured in 15 hierarchical domains (three bed, three chair, four static balance, two walking and three dynamic balance items), each measured on a two (able/unable) or three (able/partial/unable) point scale. It takes a trained person ten minutes to administer the test in elderly patients. The raw score is converted to an interval score.

#### Short Physical Performance Battery

The SPPB is composed of three tasks: a hierarchical balance task, a short walk at normal speed, and five repetitive chair stands. Low scores in the SPPB have predictive value for a wide range of health outcomes: mobility loss, disability, hospitalization, length of hospital stay, nursing home admission, and death [72-75]. Normative values of SPPB have been published for representative populations by five-year age groups and sex [73]. The strong and consistent association with health status measures demonstrated the validity of the SPPB [76].

### Patient reported outcome measures

#### EQ-5D-3L

The EuroQol/EQ-5D-3L is a validated, generalized and standardized instrument comprising a VAS measuring self-rated health and a health status instrument, consisting of a three-level response (no problems, some problems and extreme problems) for five domains related to daily activities; (i) mobility, (ii) self- care, (iii) usual activities, (iv) pain and discomfort and (v) anxiety and depression. Responses to the health status classification system are converted into an overall score using a published utility algorithm for the Dutch population [77]. A respondent’s EQ-VAS gives self-rated health on a scale where the endpoints are labeled ‘best imaginable health state’ (100) and ‘worst imaginable health state’ (0).

#### Short Form-12

The SF-12 is the abbreviated version of the original SF-36 and scores quality of life in two domains, physical and mental health. It has shown good correlation with the SF-36 and proved valid in many conditions, amongst is orthopedic surgery [78].

### Complications

The surgeon monitors all complications that may occur throughout admission, in general or cryotherapy-related. After discharge the coordinating investigator verifies if all adverse events are registered and recorded on the appropriate CRF. In case of serious adverse events relating to the treatment patients will no longer receive treatment but will remain in the study for follow-up, if feasible. Severe leakage i.e. multiple bandage swaps daily is noted and registered as an adverse event. Furthermore an intragroup analysis of vital signs is made to determine possible central effects of the administered therapy. Finally, at the outpatient visit patients are assessed for adverse events that may have occurred during rehabilitation. Additional to the patient questionnaire (Table 5), intervention patients are asked about their experiences with CFC therapy. An additional insurance is taken out to financially compensate participants if applicable. Adverse events are tabulated and reported in the final manuscript.

### Feasibility

At the end of the study nursing staff will complete questionnaires specifically drafted for this study (Table 6) and the booklet of prevalent technical problems will be evaluated. A close out visit will be planned in which recommendations for further improvement of the machine will be discussed. Treatment failure i.e. discontinuation of treatment is reported and provided with reasons e.g. discomfort.

**Table 6:** Nursing staff questionnaire

1. Was the GRS hip/groin wrap technically easy to apply?	A	B	C	D	E
2. Was the GRS hip/groin wrap easy to apply to postoperative hip fracture patients?	A	B	C	D	E
3. Did you apply the GRS hip/groin wrap alone?	Y				N
4. Was the control unit easy to operate?	A	B	C	D	E
5. If the GRS works do you think that the application (4 times 30 min a day) is feasible in daily practice?	A	B	C	D	E
6. Were you able to administer all the treatments that were required?	A	B	C	D	E
7. Would you recommend cryocompression therapy to patients?	A	B	C	D	E
8. Do you think patients recovered faster because of cryocompression therapy?	A	B	C	D	E
9. Should the cryocompression therapy be apart of standard hip fracture treatment?	A	B	C	D	E
10. Can you briefly describe what you think are advantages of cryocompression therapy?	Open text				
11. Can you briefly describe what you think are disadvantages of cryocompression therapy?	Open text				

*GRS* Game Ready system

A: agree completely; B: agree; C: neutral; D: disagree; E: disagree completely

Y: yes; N: no

## Statistics

### Analysis of outcome parameters

All statistical analyses will be computed using the SPSS statistical package (IMB SPSS, Inc., Release 20.0.0.0, 64-bit edition). Statistical analysis will be performed according to the intention-to-treat principle. Baseline characteristics will be described in accordance with CONSORT guidelines [41] using means and standard deviation in case of normal distribution, and medians and interquartile ranges otherwise. Continuous variables will be checked for normality, visually and by using the Shapiro Wilk test. Our primary analysis focuses on the differences in NRS-pain scores between the study groups 24 h postoperative and will be analyzed by use of Student’s T or Mann–Whitney U tests in case of skewed distribution. To assess treatment effect over time, a mixed model analysis for repeated measures will be performed. This model allows missing data and adjustment for serious confounders and interactions. In case of skewed distributions and outliers non-parametric variants will be used (e.g. Mann Whitney U or Friedman tests). Secondary continuous outcome measures (EQ-5D-3L, SF-12, DRS-R-98 and hemoglobin) will be compared by use of Student’s t or Mann Whitney U tests. Ordinal variables will be analyzed using the Mann–Whitney test. Chi-squared tests will be performed in case of categorical variables. In case of important confounding, analysis will be adjusted to correct for these factors (by use of multiple regression analysis, linear or logistic). A *p*-value of < 0.05 will be considered statistically significant.

### Sample size calculation

Sample size calculation is based on the primary outcome measure, NRS-pain at 24 h postoperatively. Subsequently, a mean NRS-pain score for postoperative day one was drawn from the Spaarne Gasthuis electronic patient dossier digital databanks and a standard deviation of 2.2 was calculated for pooled internal fixation and (hemi) arthroplasty for cervical and peritrochanteric fractures. The minimal clinical relevant difference for NRS-pain is 1.3 out of 10 [53]. The alpha was set at 5 % with a power of 90 % due to anticipated heterogeneity in regard to operating techniques, general care protocols but predominantly in regard to pain protocols between the participating centers (Table 2). An expected NRS failure rate of 10 % is anticipated together with expected missing data a drop out of 22.3 % is computed. The total number of included patients will be 160.

## Dissemination policy

The trial outcome will be published as an international peer-reviewed article. Any modifications to the protocol which may impact on the conduct of the study, potential benefit of the patient or may affect patient safety, including changes of study objectives, study design, patient population, sample sizes, study procedures, or significant administrative aspects will require a formal amendment to the protocol. Such amendment will be agreed upon by the REC/IRB and will be reported to www.trialregister.nl and to this journal.

## Discussion

This multicenter study will evaluate the efficacy of intermittent application of continuous-flow cryocompression therapy administered in the first 72 postoperative hours after hip fracture surgery. Due to the multicenter design of the study the results can easily be translated to the general care of hip fracture patients. Conversely, some inequality in local hospital pain protocols is present but conversion through accepted algorithms [59, 60] make comparison feasible. Spinal anesthetized patients could have advantages over the general anesthesia group in the acute hours post-surgery in regard to pain scores and analgesic use since the effect of the latter ends immediately after surgery in contrast to the former that takes a few hours to wear off completely.

The varying types of fractures have different characteristics and demand a different surgical approach. Peritrochanteric fractures quite often have more extensive bony trauma and less soft-tissue trauma, and after surgical stabilization with a DHS or IMHN fracture micro-motion leads to prolonged and greater dynamic pain [6]. Conversely, in (hemi) arthroplasty the fracture-site is removed completely at the expense of increased (surgical-induced) soft-tissue trauma. Hence the efficacy of CFC therapy is likely to vary in patients with varying extent of soft-tissue trauma. Therefore stratification is implemented according to type of surgery that divides patients with greater surgical trauma ((hemi) arthroplasty) and lesser surgical trauma (cannulated screws, DHS, IMHN).

By nature, delirium is known to fluctuate over the day and a single DRS-R-98 measurement might not reach sufficient sensitivity for diagnosis. However with longitudinal follow-up of three times daily DOS-scores this problem is overcome for the most part. Delirium severity is assessed with more information gathered from family and personnel, over the past 24 h thereby making assessment adequate.

Currently three of the four participating hospitals have a geriatric trauma unit, in one of the hospitals with a geriatric trauma unit the care of the surgical geriatric patient is transmitted to the geriatrician in full except for wound assessments and related care. At this ward all patients receive prophylactic haloperidol 1 mg daily, and more if needed. At the hospital without a geriatric trauma unit a geriatrician is not readily available and unable to attend the clinic on a daily basis. Confounding of secondary outcomes as delirium, psychotropic medication and functional outcome can therefore not be ruled out.

When compared to the preoperative values, hemoglobin levels in collected blood samples at postoperative day one are usually much lower than one would expect from the observed blood loss during surgery. This gap called ‘the hidden blood loss’ is thought to arise from, among others gastro-intestinal bleeding [22, 79]. The amount of (hidden) blood loss is more severe when oral anticoagulants or thrombocyte aggregation inhibitors compromise a patient’s hemostasis and this loss may continue throughout the early postoperative period. Hemoglobin measured at postoperative day one is most likely, as opposed to the preoperative values, significantly diluted, thereby underestimating the actual hemoglobin concentration. Combined with the possibility of hidden blood loss throughout the hospital stay, care has to be taken when comparing the value of postoperative day one to day three.

The functional outcome assessment of patients who are not allowed to bear weight at the time of discharge is postponed to the outpatient clinic. At this visit they are allowed to bear weight for the first time in weeks. Testing directly after weight bearing for the first time can introduce confounding.

Finally, care has to be taken to interpret the allocation to rehabilitation facilities. For instance patients who have sufficient family members who can help out at home are more likely to be discharged home with or without homecare rather than be assigned to a rehabilitation clinic or nursing home with rehabilitation facilities.

## Conclusion

The present study will provide evidence for the efficacy of continuous-flow cryocompression therapy applied after hip fracture surgery. Due to the duplicate trauma this condition encompasses these patients, who are generally aged 70 years and over, are expected to benefit most. Furthermore treatment feasibility is assessed and consequently, recommendations are made about which settings are best to employ.

### Ethics and consent to participate

Permission has been obtained from the 'Medisch Ethische Toetsingscommissie Noord-Holland' regional medical ethics committee (NL45657.094.14) and the trial is registered at www.trialregister.nl on the 23^rd^ of August 2013 with trial number: “NTR4152”. Patients with an intra or extracapsular hip fracture who meet the inclusion criteria and without apparent exclusion criteria (Table 1) are informed about the study both orally and in writing by the assessing physician e.g. the local ward doctor, staff member or consulting geriatricians. Depending on operating room capacity and patient characteristics patients will either first be transferred to the ward or will go directly to the operating room. Patients can be included pre and postoperatively within 6 h post surgery, before the first treatment starts 6 h postoperative (Fig. 1). Maximum time for consideration to participate in the study is set at 12 h. Patients approving to participate need to sign the informed consent form after which baseline characteristics are noted.

### Consent to publish

Not applicable.

### Availability of data and materials

Not applicable as this is a protocol of a study and no data are reported.

## Abbreviations

A&E: accident and emergency
ASA: American Society of Anesthesiologists
CFC: continuous-flow cryocompression
CRF: case report form
DEMMI: de morton mobility index
DHS: dynamic hip screw
DOS: delirium observation screening
DRAS: delirium risk assessment score
DRS-R-98: delirium rating scale revised 1998
EMS: elderly mobility scale
EQ-5D: EuroQol-5D
GRS: game ready system
HA: hemiarthroplasty
IQ-CODE: informant questionnaire for the cognitive decline in the elderly
IRB: institutional review board
MMSE: mini mental state examination
NMS: new mobility score
NRS: numeric rating scale
NSAID’s: non-steroidal anti-inflammatory drugs
PCA: patient controlled analgesia
PROM: patient reported outcome measure
REC: Regional ethics committee
RM: research manager
SF-12: short form-12
SF-36: short form-36
SPPB: short physical performance battery
THA: total hip arthroplasty
TKA: total knee arthroplasty
TUGT: timed up and go test
VAS: visual analogue scale
